# Dietary “Beigeing” Fat Contains More Phosphatidylserine and Enhances Mitochondrial Function while Counteracting Obesity

**DOI:** 10.34133/research.0492

**Published:** 2024-09-26

**Authors:** Yanbing Zhou, Defeng Ling, Liyi Wang, Ziye Xu, Wenjing You, Wentao Chen, Qiuyun Nong, Teresa G. Valencak, Tizhong Shan

**Affiliations:** ^1^ College of Animal Sciences, Zhejiang University, Hangzhou, Zhejiang 310058, China.; ^2^ Key Laboratory of Molecular Animal Nutrition (Zhejiang University), Ministry of Education, Hangzhou, Zhejiang 310058, China.

## Abstract

Activation of mitochondrial function and heat production in adipose tissue by the modification of dietary fat is a promising strategy against obesity. However, as an important source of lipids for ketogenic and daily diets, the function of fats extracted from different adipose tissue sites was largely unknown. In this study, we illustrated the function of fats extracted from adipose tissues with different “beigeing” properties in the ketogenic diet and identified lipid profiles of fats that facilitate energy expenditure. We found that the anti-obesity effect of ketogenic diets was potentiated by using “beigeing” fat [porcine subcutaneous adipose tissue (SAT)] as a major energy-providing ingredient. Through lipidomic analyses, phosphatidylserine (PS) was identified as a functional lipid activating thermogenesis in adipose tissue. Moreover, in vivo studies showed that PS induces adipose tissue thermogenesis and alleviates diet-induced obesity in mice. In vitro studies showed that PS promotes UCP1 expression and lipolysis of adipocytes. Mechanistically, PS promoted mitochondrial function in adipocytes via the ADCY3-cAMP-PKA-PGC1α pathway. In addition, PS-PGC1a binding may affect the stability of the PGC1α protein, which further augments PS-induced thermogenesis. These results demonstrated the efficacy of dietary SAT fats in diminishing lipid accumulation and the underlying molecular mechanism of PS in enhancing UCP1 expression and mitochondrial function. Thus, our findings suggest that as dietary fat, “beigeing” fat provides more beneficial lipids that contribute to the improvement of mitochondrial function, including PS, which may become a novel, nonpharmacological therapy to increase energy expenditure and counteract obesity and its related diseases.

## Introduction

Obesity and obesity-associated comorbid diseases, such as type 2 diabetes mellitus, cardiovascular disease, and cancer, have reached pandemic levels worldwide and are major threats to human life [1]. White adipose tissue (WAT) is the major energy storage in mammals and is distributed in several depots throughout the body [2]. Excess WAT expansion due to accumulated energy storage drives the progression of obesity [3]. Apart from their critical role for storing energy, the 2 other adipose tissue types serve as hubs for heat generation. Brown adipose tissue (BAT) dissipates energy through nonshivering thermogenesis releasing energy in the form of heat [3,4]. Related beige or “brite” adipocytes, which partially have BAT features, are formed in WAT upon sympathetic nerve innervation, chemical (or hormonal) stimulation, exercise training, or cold exposure [2,5]. Both brown and beige adipocytes contain many mitochondria and highly express Uncoupling protein 1 (UCP1), which is key for regulating their function in energy homeostasis and fat deposition [6]. As the functional properties of energy storage and expenditure are intrinsically connected, any activation of BAT and transformation of white adipocytes into beige adipocytes are promising strategies combatting the global obesity epidemic [1,2].

Several nutritional strategies regulating thermogenesis and lipid metabolism in adipose tissue have been proposed previously. In the past 2 decades, many nutrients promoting energy expenditure of adipose tissue have been discovered, e.g., amino acids, fatty acids, and plant-derived compounds [7–9]. On the other hand, sensible selection of carbohydrates, proteins, and fatty acids in the daily diet is also an effective strategy to control obesity [10]. Among the available strategies out there in the field of dietary restriction, the ketogenic diet (KD), composed of high fat (>60% of energy), adequate protein, and few carbohydrates (<5% of energy), has been established as nonpharmacological treatment for various types of epilepsy recently and has emerged as an alternative option for obesity management [11,12]. Many previous studies have tried to answer whether the fat source and fatty acid composition of the KD influence its efficacy as an anti-obesity strategy [13,14]. As a major part of fat, there are some lipid species that regulate adipose tissue thermogenesis and energy balance, such as plasmalogen [15,16], cardiolipin [17], lysophosphatidic acid (LPA), and fatty acid esters of hydroxy fatty acids (FAHFAs) [18,19]. Moreover, the role of adipose tissue as major metabolic and endocrine organ has received much attention. Lipid composition of adipose tissue is modified when meeting energetic needs, which vary greatly according to the depot-specific adipogenic potential, metabolic characteristics, and “beigeing” capacity of the adipose tissue depot [3,20,21]. Recent studies have mainly focused on physiological lipid function as a component of adipose tissue for maintaining metabolic homeostasis. Whether or not lipid composition of adipose tissue relates to the “beigeing” capacity could have beneficial effects on metabolism in the KD and certainly is worth being studied in detail.

Here, we compared the effects of fats extracted from SAT and VAT, 2 representative adipose tissues with different “beigeing” susceptibility, for regulating lipid accumulation in a mouse model with diet-induced obesity (DIO) being exposed to a KD. Interestingly, we can show that SAT fat is more efficiently activating BAT function and better alleviating hepatic steatosis in DIO mice compared to VAT fat. Through integrated lipidomic analysis, phosphatidylserine (PS) was identified as a promising lipid that exogenously increases UCP1 expression and attenuates DIO. In vitro and in vivo studies have demonstrated that PS stimulates expression of adenylate cyclase 3 (ADCY3) and up-regulates intracellular cAMP concentration, which activates PKA-PGC1α signaling and facilitates mitochondrial function. In addition, PS regulates PGC1α protein stability by lipid–protein binding and might induce thermogenesis in adipocytes. Our findings highlight a novel role of PS in modulating thermogenesis in adipose tissue and put forward PS as nonpharmacological tool to counteract obesity and its related metabolic conditions.

## Results

### SAT fat enhances BAT function of KD-fed DIO mice

**Fig. 1.**
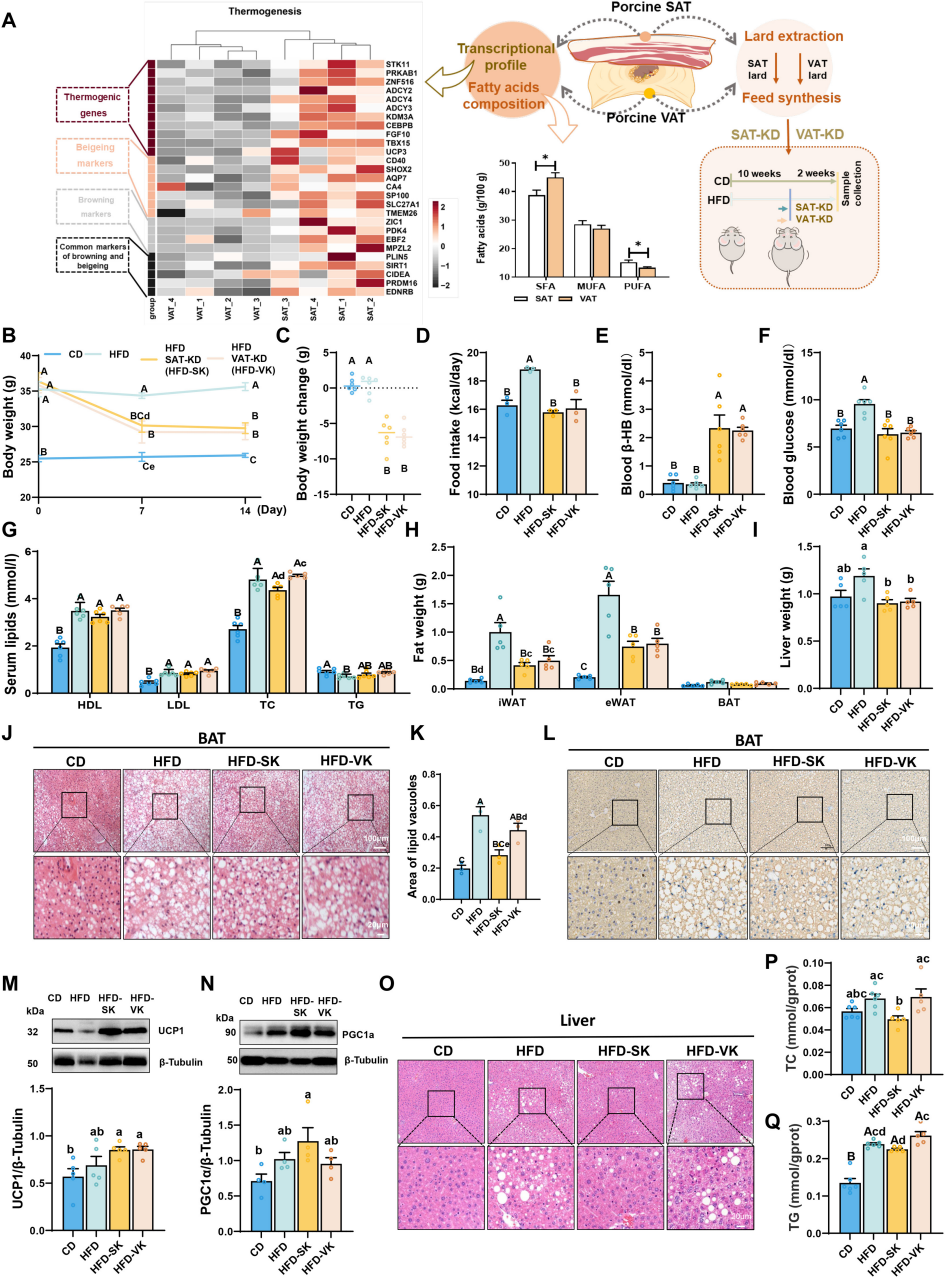
SAT-Fat-based KD decreased lipid deposition in BAT and liver of DIO mice. (A) Scheme of the experimental process. The heatmap depicted the thermogenesis-associated DEGs in subcutaneous adipose tissue (SAT) and visceral adipose tissue (VAT) of pigs. Bar graph displayed the contents of total SFAs, MUFAs, and PUFAs in porcine SAT and VAT. SFAs, saturated fatty acids; MUFAs, monounsaturated fatty acids; PUFAs, polyunsaturated fatty acids containing 2 or 3 to 6 double bonds. The data are presented as the means ± SEM. *P < 0.05, **P < 0.01, 2-tailed Student’s *t* test. (B) Body weights of SAT-KD-fed mice, VAT-KD-fed mice, HFD-fed mice, and CD-fed mice on days 0, 7, and 14 (6 mice per group). (C) Time courses of body weight changes of mice fed the respective diets for 14 days. (D) Food intake. (E) Blood β-HB and (F) glucose in mice 3 h after meal. (G) Serum lipid contents. HDL, high-density lipoprotein cholesterol; LDL, low-density lipoprotein cholesterol; TC, total cholesterol; TG; triglyceride. (H) Weight of fat tissues and (I) liver tissue. iWAT, inguinal white adipose tissue; eWAT, epididymal adipose tissue; BAT, brown adipose tissue. (J) Representative H&E staining BAT from 4 groups. Scale bars, 100 μm. (K) Area of lipid vacuoles in BAT from 4 groups (*n* = 3). (L) Representative UCP1 immunostaining of BAT from 4 groups. Scale bars, 100 mm. (M) Protein levels of UCP1 and (N) PGC1α in BAT. (O) Representative H&E staining of liver sections. (P) TG and (Q) TC contents in the liver of mice (*n* = 5 to 6). The data are presented as means ± SEM. One-way ANOVA with Tukey’s test. Groups with different superscript lowercase letters were significantly different (*P* < 0.05), and groups with different superscript uppercase letters differed even more significantly (*P* < 0.01).

To directly investigate the “beigeing” properties of different porcine adipose tissue sites, we analyzed the RNA sequencing (RNA-seq) data of SAT and VAT from our previous study to map the transcriptional differences [22]. The expression of thermogenesis regulating genes *PRKAB1*, *ZNF516*, and *UCP3* and “beigeing” markers *PRDM16*, *ZIC1*, and *PDK4* were significantly up-regulated in porcine SAT, which indicated that porcine SAT was more active in lipid metabolism and had higher “beigeing” activity than VAT (Fig. 1A). Next, we determined fatty acid composition of porcine fats and found that VAT contains more saturated fatty acids (SFAs), while SAT contains more polyunsaturated fatty acids (PUFAs) (Fig. 1A). Then, fats were extracted using the hydro-enzymatic method [23] for the synthesis of KD foodstuffs (Fig. 1A). We performed a short-term KD-feeding experiment in DIO male C57BL/6 mice and found that KD-fed mice had a distinct decrease in body weight and food intake compared to HFD-fed mice (Fig. 1B to D). KD-fed mice had higher circulating beta-hydroxybutyric acid (β-HB) levels and lower blood glucose (Fig. 1E and F). With regard to serum lipid contents, the SAT fat-based KD (HFD-SAT-KD, HFD-SK) group had a lower serum total cholesterol (TC) content than the VAT fat-based KD (HFD-VAT-KD, HFD-VK) group (Fig. 1G). Both HFD-SV and HFD-VK mouse groups had reduced fat mass and liver weights (Fig. 1H and I). Hematoxylin and eosin (H&E) staining confirmed a KD-associated reduction in the adipocyte size of WAT (Fig. S1A and B), and we observed a reduction in the lipid vacuole area in BAT of HFD-SK mice (Fig. 1J and K). Consistent with this observation, KD increased UCP1 levels in inguinal WAT (iWAT) and BAT (Fig. 1L and M and Fig. S1C and D). The protein level of peroxisome proliferator-activated receptor gamma coactivator 1-alpha (PGC1α) was significantly increased in the BAT of HFD-SK mice compared with that of the other groups (Fig. 1N). In liver tissue, SAT-KD attenuated HFD-induced TG and TC accumulation, whereas VAT-KD aggravated hepatic steatosis (Fig. 1O to Q). Overall, the fat-based KDs reduced lipid accumulation and promoted UCP1 expression in adipose tissue of obese mice. Furthermore, SAT fat promoted PGC1α expression along with reduced lipid storage in BAT and effectively reduced serum and liver TC levels in obese mice on a KD.

### PS contributes to the beneficial effects of SAT fats in regulating lipid metabolism

**Fig. 2.**
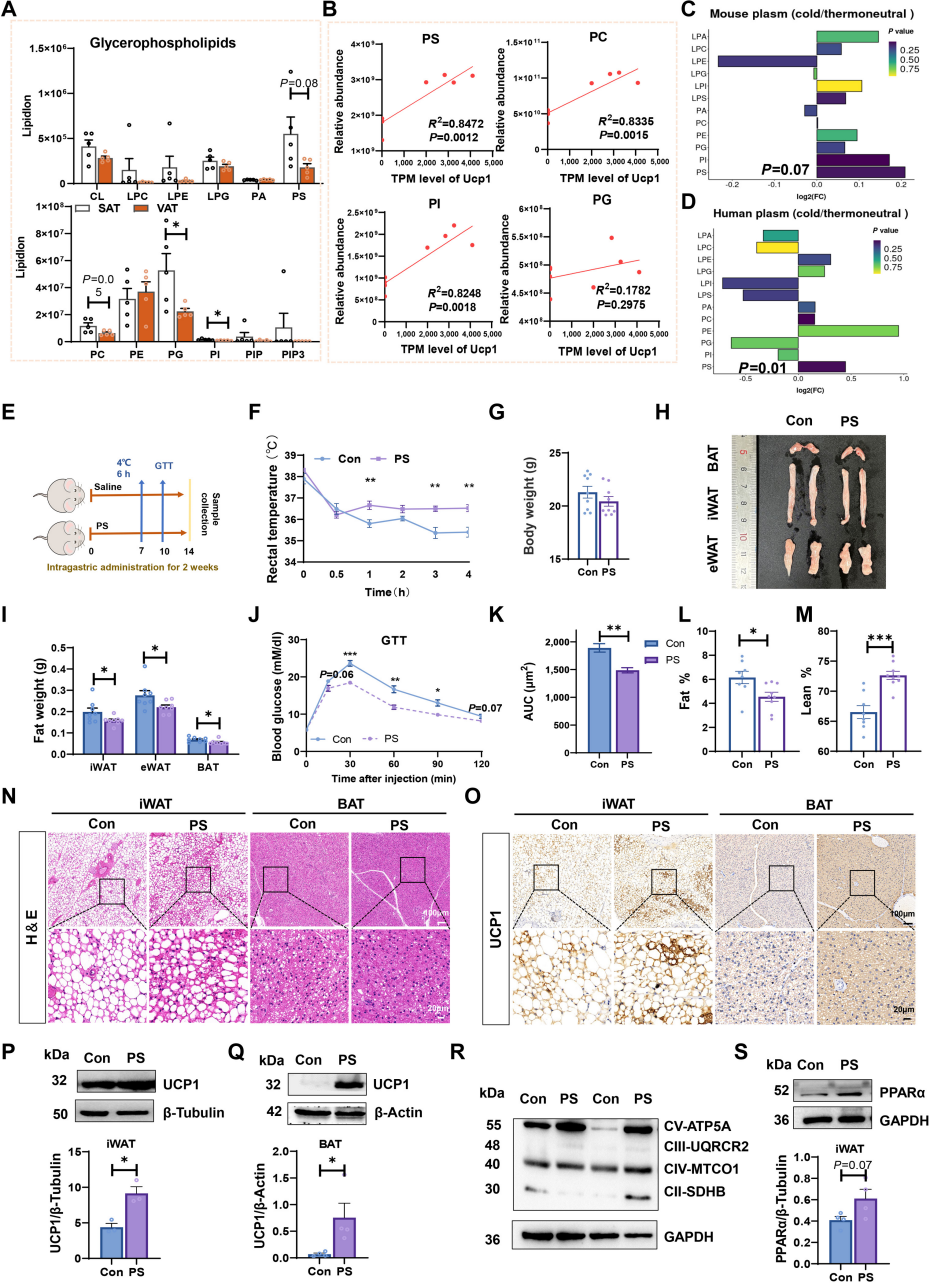
PS as specific lipid modulated lipid accumulation in adipose tissue. (A) Concentrations of GPs in SAT and VAT. LPC, lysophosphatidylcholine; LPE, lysophosphatidylethanolamine; LPG, lysophosphatidylglycerol; CL, cardiolipin; PA, phosphatidic acid; PS, phosphatidylserine; PC, phosphatidylcholine; PE, phosphatidylethanolamine; PG, phosphatidylglycerol; PI, phosphatidylinositol; PIP, phosphatidylinositol phosphate; PIP3, phosphatidylinositol triphosphate. The data are presented as the means ± SEM. *P < 0.05, **P < 0.01, 2-tailed Student’s *t* test. (B) Correlation analysis between GP content and UCP1 expression in mouse iWAT. (C) Changes in GP composition in plasma after cold exposure in mice and (D) humans. The transparency of each bar is proportional to the significance value, which is displayed as −log10 (*P* value). (E) Scheme of the experimental process. Control group, normal mice treated with Saline; PS group, normal mice treated with PS 130 mg kg^−1^ BW per day for 2 weeks. Acute cold exposure (4 °C, 6 h) was performed on day 7 (D7) and GTT was performed on D10. (F) Rectal temperature of mice after cold stimulation. (G) Body weights of mice after 2 weeks of administration. (H) Representative macroscopic images of mouse adipose tissue after 14 days of experiment. (I) Adipose tissue weights. (J) Blood glucose concentrations and (K) calculated area under the curve (AUC) during glucose tolerance tests (GTTs) performed in Con and PS male mice (*n* = 6). (L) Lean body mass and (M) body fat percentage in Con and PS mice (*n* = 9). (N) Representative H&E staining of iWAT and BAT sections. Scale bars, 100 μm. (O) Representative UCP1 immunostaining of iWAT and BAT from 2 groups. Scale bars, 100 μm. (P) The protein level of UCP1 in iWAT and (Q) BAT of Con and PS mice. (R) The protein levels of ETC (electron transport chain) complexes (ATP5A, ATP synthase, H^+^ transporting, mitochondrial F1 complex, alpha 1; UQCRC2, ubiquinol-cytochrome c reductase core protein II; MTCO1, cytochrome c oxidase I; SDHB, succinate dehydrogenase complex iron sulfur subunit B) in iWAT of Con and PS mice. (S) The protein levels of PPARα in iWAT of Con and PS mice. The data are presented as means ± SEM. *P < 0.05, **P < 0.01, ***P < 0.001, 2-tailed Student’s *t* test.

To better understand the effects of SAT and VAT for promoting BAT activation and regulating lipid accumulation, we analyzed lipid composition of SAT and VAT by mass spectrometry-based lipidomic analysis. We found a significant increase in phosphatidylglycerol (PG) and phosphatidylinositol (PI) and an increasing trend in PS (*P* = 0.08) and phosphatidylcholine (PC) (*P* = 0.05) in the GPs pool of SAT (Fig. 2A). Previous studies have found remodeling of GPs in adipose tissue during thermogenesis [20,24]. By analyzing published data, we found that PC, PI and PS were highly correlated with cold-induced *Ucp1* expression in mouse iWAT [24] (Fig. 2B). Among them, only PS was highly elevated in both mice and human plasm after cold exposure [25] (Fig. 2C and D). By combining the lipidomic results with previous findings, we identified PS as a candidate that plays a role for regulating thermogenesis in adipose tissue.

To determine the function of PS in lipid metabolism of adipose tissue, we performed a PS administration test in female and male C57BL/6 mice (Fig. 2E). After 7 days of continuous administration, the core temperature of PS mice was significantly higher than that observed in the Con group after 4 h at 4 °C (Fig. 2F and Fig. S2A). We also measured heat dissipation of mice before and after 6 h of cold exposure and found that short-term PS supplementation contributed to the maintenance of body temperature in mice under cold exposure (Fig. S2B and C). Although there was no significant change in body weight (Fig. S2D), we observed that iWAT and BAT were reduced in both female and male mice by PS treatment, with a trend toward a decrease in epididymal fat (eWAT) in male mice (*P* = 0.08) (Fig. S2E). Mice exhibited profound browning of iWAT (Fig. S2F and G). We then administered PS to male mice for 14 days and found that there was still no significant difference in body weights between the 2 groups (Fig. 2G). However, iWAT, eWAT, and BAT were significantly reduced due to PS treatment (Fig. 2H and I). Consistently, PS-treated mice maintained better glucose tolerance (Fig. 2J and K). Notably, PS-treated mice demonstrated a lower body fat percentage and a higher lean body mass in comparison to the control group (Fig. 2L and M). PS-treated mice had large regions of multilocular adipocytes, accompanied by higher expression of UCP1 in iWAT and BAT (Fig. 2N to Q). The protein levels of mitochondrial protein complexes (ATP5A (V) and MTCO1 (IV)) and peroxisome proliferator-activated receptor α (PPARα) were found to be remarkably up-regulated in iWAT of PS-treated mice (Fig. 2R and S). The above results indicated that PS might induce iWAT browning, enhance BAT function, and affect mitochondrial function of mice.

### PS alleviated high-fat DIO in mice

**Fig. 3.**
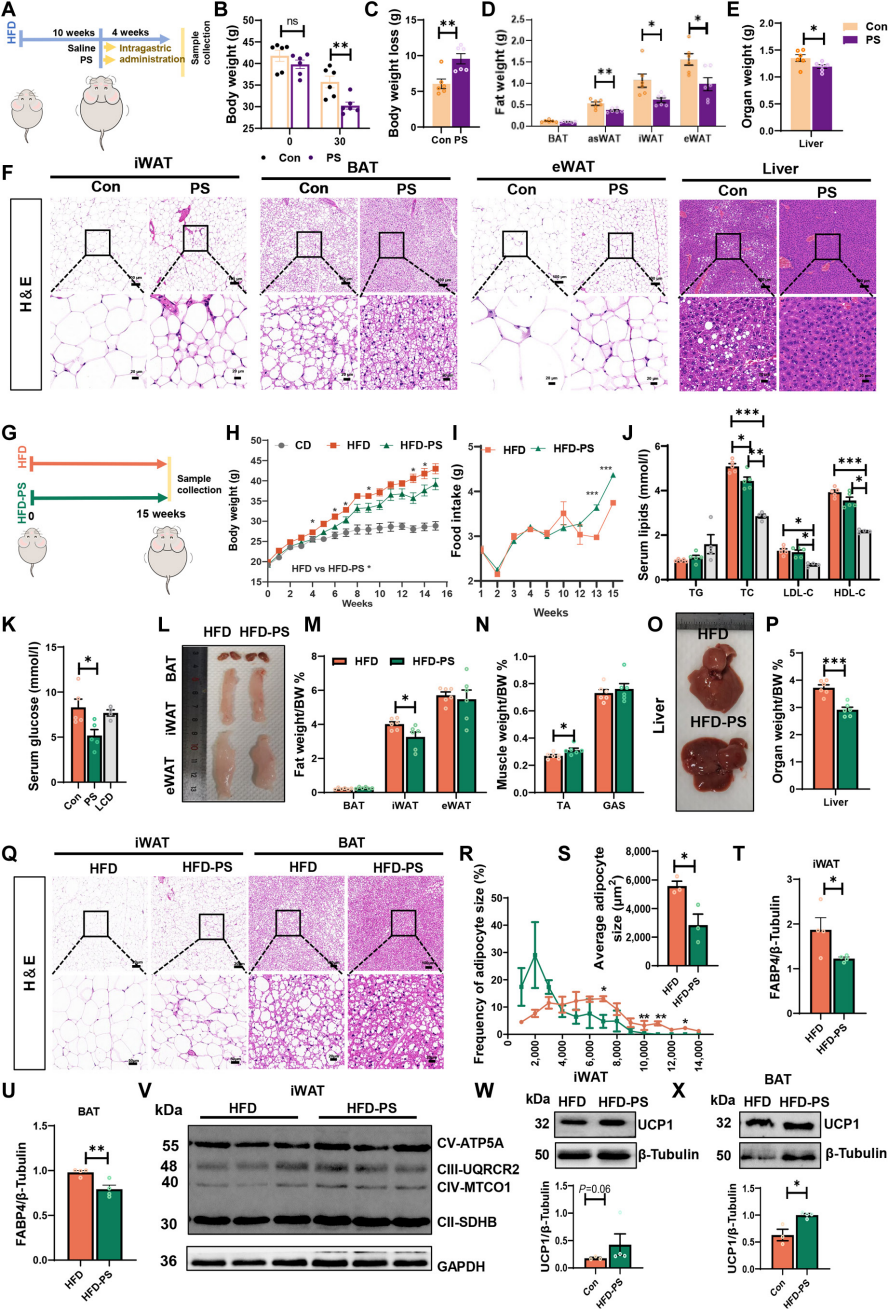
PS alleviated HFD-induced lipid deposition. (A) Scheme of the experimental process. HFD group, HFD fed obese mice treated with Saline; PS group, HFD fed obese mice treated with PS 130 mg kg^−1^ BW per day for 4 weeks. (B) Body weights of DIO mice at day 0 and day 30 under normal saline or PS treatment (6 mice per group). (C) Body weight change. (D) Adipose tissue weight and (E) liver weights from the 2 groups. The error bars represent the SEM. *P < 0.05, **P < 0.01, 2-tailed Student’s *t* test. (F) Representative H&E staining of iWAT, eWAT, BAT, and liver sections. Scale bars, 100 μm. (G) Scheme of the experimental process. HFD group, mice fed an HFD for 15 weeks; HFD-PS group, mice fed an HFD that contained 1% PS for 15 weeks. (H) Body weight changes. (I) Food intake. (J and K) Levels of serum lipids and glucose. (L) Representative macroscopic pictures of adipose tissues. (M) Fat index = fat weight/body weight × 100. (N) Muscle index = muscle weight/body weight × 100. (O) Representative macroscopic pictures of livers. (P) Organ index = fat weight/body weight × 100. (Q) Representative H&E staining of BAT and iWAT sections. The scale bar is marked on the picture. (R) Frequency of adipocyte size in mouse iWAT sections (*n* = 3). (S) Average adipocyte size of iWAT sections (*n* = 3). (T) Protein level of FABP4 in iWAT and (U) BAT. (V) The protein levels of ETC (electron transport chain) complexes in iWAT of HFD and HFD-PS mice. (W) The protein levels of UCP1 in iWAT and (X) BAT HFD and HFD-PS mice. The data are presented as means ± SEM. *P < 0.05, **P < 0.01, ***P < 0.001, 2-tailed Student’s *t* test.

To confirm the observation that PS induced browning of WAT and activated BAT, we performed a PS supplementation study in DIO mice (Fig. 3A). After 4 weeks, we measured the body weights of mice and found that daily administration of PS significantly decreased mouse body weights (Fig. 3B and C). The weights of WAT and liver tissue were significantly decreased in the PS group (Fig. 3D and E). Consistently, adipocyte size and lipid vacuoles were reduced by PS administration (Fig. 3F).

To assess the effects of long-term PS supplementation on fat accumulation, mice received supplementation with 1% PS continuously for 15 weeks (Fig. 3G). Expectedly, body weight gain in HFD-fed mice was significantly elevated compared to that in the PS-treated group (Fig. 3H). PS-treated mice exhibited higher food intakes in the last 2 weeks of the experiment (Fig. 3I). PS intervention markedly decreased serum TC and glucose levels compared to the HFD group (Fig. 3J and K). Consistent with the macroscopic pictures and weights of tissues and organs (Fig. 3L to P), H&E staining confirmed that PS alleviated HFD-induced lipid storage in adipose and liver tissues (Fig. 3Q and Fig. S3A). In adipose tissue, PS induced an obvious decrease in the frequency of large adipocytes (>7,000 μm) and the average adipocyte size (Fig. 3R and S). The expression of FABP4 was decreased in iWAT and BAT due to PS treatment (Fig. 3T and U). In the liver, both TG and TC levels were decreased following PS treatment (Fig. S3B and C). The protein levels of mitochondrial protein complexes (MTCO1 (IV)) and UCP1 were found to be significantly up-regulated in adipose tissues of HFD-PS mice (Fig. 3V to X). These findings indicated that treatment with PS can both prevent and mitigate the accumulation of lipids in the adipose tissue and liver of obese mice.

### PS promoted lipolysis and UCP1 expression to reduce fat accumulation

**Fig. 4.**
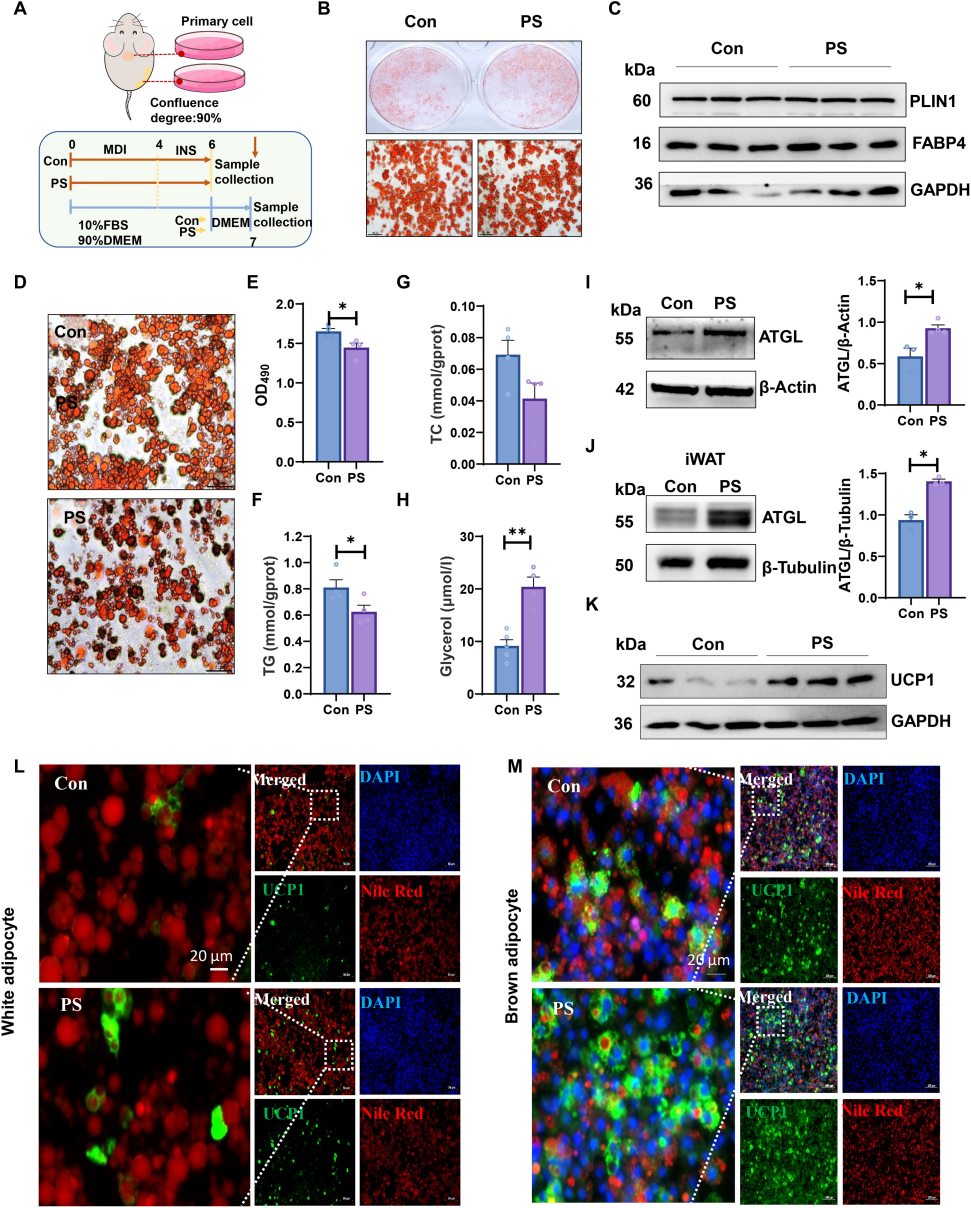
PS promoted lipolysis to reduce lipid accumulation in white and brown adipocytes. (A) Scheme of the experimental process. (B and C) Mouse white pre-adipocytes were treated with 10 μM PS (DMEM) or DMSO (DMEM) during adipogenic differentiation. (B) Oil Red O staining of intracellular lipid droplets. Scale bar, 100 μm. (C) Protein levels of PLIN1 and FABP4 in Con and PS-treated adipocytes. (D and E) Differentiated adipocytes were treated with 10 μM PS (DMEM) or DMSO (DMEM) for 24 h. (D) Intracellular lipid droplets stained with Oil Red O dye after lipolysis were examined by light microscopy (scale bar, 100 μm) and (E) the absorbance of the extracted Oil Red O dye in isopropyl alcohol was measured at 490 nm (*n* = 3). (F) TG, (G) TC contents in whole cell lysates and (H) glycerol content in culture medium. (I) Protein levels of ATGL in adipocytes and (J) iWAT of mice with or without PS treatment. (K) Protein level of UCP1 in PS-treated and Con adipocytes. (L) Differentiated white or (M) brown adipocytes treated with 10 μM PS or DMSO for 24 h were fixed and stained with a UCP1 antibody conjugated to Alexa Fluor 647 (green). Lipid droplets were stained with Nile Red (red). Nuclei were stained with DAPI (blue). Images were taken with a fluorescence microscope. Scale bar, 50 μm. The data are presented as means ± SEM (*n* = 3 to 4). *P < 0.05, 2-tailed Student’s *t* test.

To elucidate the role of PS in vitro, we isolated the stromal vascular fraction (SVF) cells from iWAT and BAT of mice and examined the effects of PS on adipogenic differentiation and the lipolysis process (Fig. 4A). Oil Red O staining and Western blot results suggested that PS treatment did not influence lipid accumulation in preadipocytes at the differentiation stage (Fig. 4B and C). However, we observed that PS markedly promoted lipolysis in differentiated adipocytes, which was characterized by a reduction in lipid droplet aggregation and a decrease in triglyceride (TG) levels, coincident with an elevation in the release of glycerol (Fig. 4D to H). Consistently, the protein levels of ATGL were significantly increased in adipocytes and iWAT of mice due to PS supplementation (Fig. 4I and J). In addition, PS-treated white adipocytes and brown adipocytes showed significantly elevated UCP1 expression (Fig. 4K to M). Collectively, the above observations suggest that PS supplementation increased UCP1 expression and facilitated lipolysis in adipocytes.

### PS promoted mitochondrial function and augmented PGC1α level

**Fig. 5.**
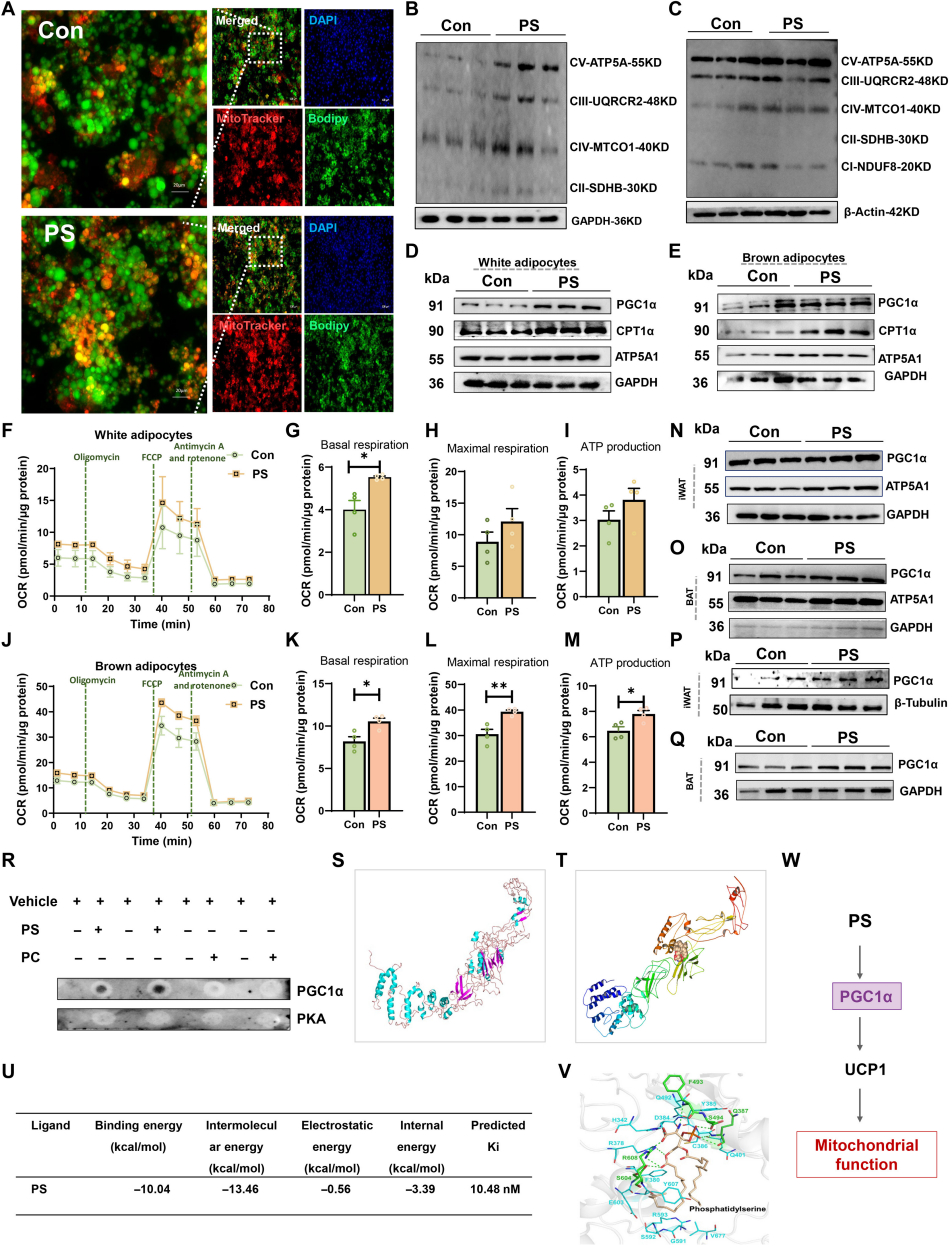
PS promoted the function of adipocyte mitochondria to facilitate the lipolysis process. (A to M) Differentiated cells were treated with 10 μM PS (DMEM) or DMSO (DMEM). (A) Mitochondrial staining (MitoTracker Red CMXRos) of white adipocytes, 24 h after treatment. Scale bar, 100 μm. (B and C) Protein levels of ETC (electron transport chain) complexes (ATP5A, ATP synthase, H^+^ transporting, mitochondrial F1 complex, alpha 1; UQCRC2, ubiquinol-cytochrome c reductase core protein II; MTCO1, cytochrome c oxidase I; SDHB, succinate dehydrogenase complex iron sulfur subunit B;NDUFB8, ubiquinone oxidoreductase subunit B8) in white adipocytes treated with PS or DMSO for 6 h (B) or 24 h (C). (D) Protein levels of PGC1α, CPT1α, and ATP5A1 in iWAT or (E) BAT after 24 h of treatment. (F to I) An OCR assay was used to observe basal and maximal mitochondrial respiratory function in differentiated white adipocytes and (J to M) brown adipocytes after 24 h of treatment (*n* = 4). (N) Protein levels of PGC1α, and ATP5A1 in iWAT and (O) BAT of PS (or saline)-administered normal mice. (P) Protein levels of PGC1α in iWAT and (Q) BAT of PS (or saline)-treated DIO mice. (R) Lipid dot-blot assays of protein extracts from murine BAT treated with PS and used to detect PS binding ability with PGC1α and PKA. (S) Protein structure of PGC1α. (T) PS binding to PGC1α in 3 dimensions. (U) The molecular docking energy score and predicted inhibitory activity of PS and PGC1α. (V) 3D binding pattern of PS with PGC1α; the green dashed line shows hydrogen bonding. (W) Schematic diagram of PS affecting mitochondrial function of adipocytes. The data are presented as means ± SEM (*n* = 5). *P < 0.05, **P < 0.01, 2-tailed Student’s *t* test.

To understand the mechanism by which PS affected *Ucp1* expression, we investigated the biological role of PS in the mitochondrial function of adipocytes. We used MitoTracker staining to examine mitochondrial density and found that MitoTracker Red CMXRos was markedly higher in PS-treated cells (Fig. 5A). Consistent with the staining results, PS supplementation enhanced adipocyte expression of complexes CV (ATP5A) and CIV (MTCO1) (Fig. 5B and C). PGC1α and carnitine palmityl transferase 1-alpha (CPT1α) were significantly up-regulated in differentiated white and brown adipocytes due to PS treatment (Fig. 5D and E). To directly assess the effect of PS on mitochondrial respiratory function, we measured the mitochondrial oxygen consumption rate (OCR) of differentiated iWAT and BAT adipocytes by using a Seahorse XF96 Extracellular Flux Analyzer (Fig. 5F to M). Compared to differentiated iWAT adipocytes, differentiated BAT adipocytes exhibited a more sensitive response to PS stimulation, as visible from significantly elevated respiration at both baseline and FCCP-induced respiration level (Fig. 5K and L). We found that PGC1α was significantly up-regulated due to PS administration in adipose tissue of both normal and obese mice (Fig. 5N to Q). In addition, lipids could contribute to the structural stability and biological function of protein through protein–lipid interactions [17,26]. Thus, we tested whether PS maintained high levels of PGC1α in adipocytes through direct interactions. We performed a lipid–protein overlay assay and found that PS strongly interacted with PGC1α (Fig. 5R). To further explore the critical residues of PGC1α responsible for PS binding, we performed ab initio modeling of PGC1α and computed docking models of PS in PGC1α, revealing that PS bound predominantly to the hydrophobic cavity formed in the middle structural domain of the PGC1α protein (Fig. 5S and T). AutoDock predicted that PS binds well to the PGC1α protein (Fig. 5U).The major contributors to the PGC1α–PS interaction are predicted to be the amino acid residues Ser604 and Arg608, forming a hydrogen bond located in the nuclear localization signal (NLS) region, and this region is associated with the stability of the PGC1α protein (Fig. 5V) [27]. These findings suggest that PS enhances mitochondrial function with increased expression and stability of PGC1α, while promoting lipolysis and UCP1 expression (Fig. 5W).

### PS activates ADCY3-cAMP-PKA signaling pathway to enhance mitochondrial function

**Fig. 6.**
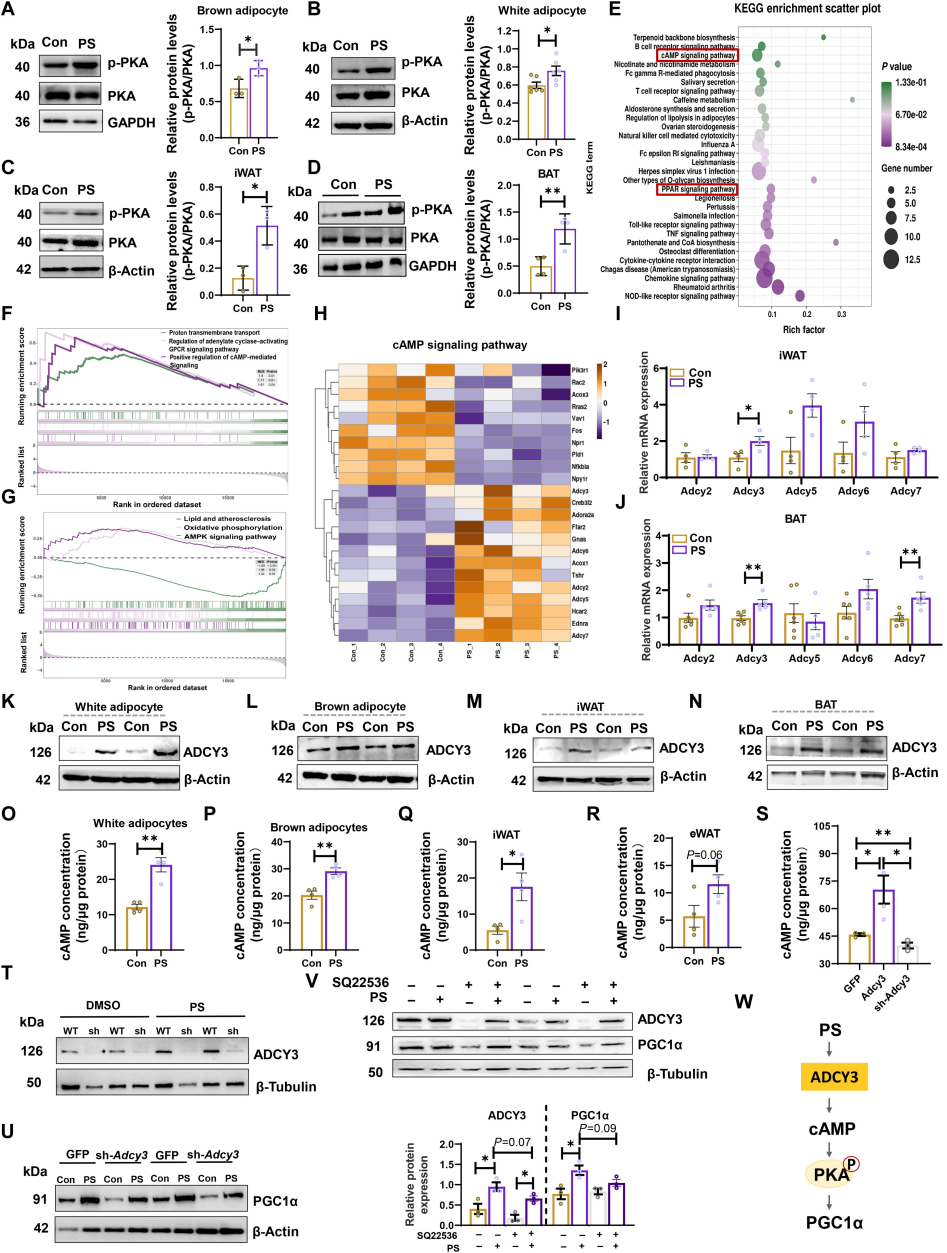
PS-enhanced mitochondrial function through ADCY3-cAMP-PKA-PGC1α signaling pathway. (A) Phosphorylation levels of PKA in brown adipocytes (*n* = 3) and (B) white adipocytes (*n* = 6) after 24 h PS (or DMSO) treatment. (C) Phosphorylation levels of PKA in iWAT (*n* = 3) and (D) BAT (*n* = 4) of PS in DIO mice. (E) Functional enrichment analyses of RNA-seq data of PS-treated white adipocytes using KEGG pathway. (F) GO and (G) KEGG enrichment pathways based on GSEA of RNA-seq data of PS-exposed white adipocytes of mice. (H) Heatmaps of the PS-induced enrichment of genes involved in the cAMP signaling pathway. (I) Relative expression of *Adcy* genes in PS- treated white adipocyte and (J) PS-administered normal mice iWAT at the mRNA level. (K) Expression of ADCY3 protein in white adipocyte, (L) brown adipocyte, (M) iWAT, and (N) BAT of HFD-PS mice after PS treated. (O) cAMP concentration in white adipocytes, (P) brown adipocytes and (Q and R) WAT of HFD mice after PS treatment. (S) Intracellular cAMP concentration in PS treated *Adcy3* over expression 3T3-L1 cell line and *Adcy3* knockdown 3T3-L1 cell line. (T and U) Expression of ADCY3 and PGC1α in *Adcy3* knockdown 3T3-L1 cell line after PS treatment. (V) Expression of ADCY3, PGC1α in 3T3-L1 cell line after treated with 10 μM SQ22536 and 10 μM PS for 3 h. The data are presented as means ± SEM (*n* = 3 to 6). *P < 0.05, **P < 0.01, 2-tailed Student’s *t* test. (W) Schematic diagram of PS enhanced ADCY3-cAMP-PKA signaling in mouse adipocytes.

Protein kinase A (PKA) is a key pathway in the regulation of PGC1α [28]; thus, we examined the phosphorylation levels of PKA and found that it was markedly increased in differentiated adipocytes and adipose tissue of mice due to PS treatment (Fig. 6A to D). To further investigate the signaling pathway associated with PS-induced PKA-PGC1α activation, we performed RNA-seq on SVFs isolated from differentiated iWAT of mice treated with or without PS. A total of 396 differentially expressed genes (DEGs) were identified in the PS and Con groups using filter criteria of |log_2_ (fold change)| > 1.5 and *q* value < 0.05, out of which 135 were up-regulated and 261 were down-regulated (Fig. S4A). The principal coordinates analysis (PCoA) plot showed a clear separation of PS and Con (Fig. S4B). RNA-seq showed that beige adipocyte markers/cell death inducing DFFA-like effector A (*Cidea*) and fibroblast growth factor 21 (*Fgf21*) were significantly up-regulated in the PS group (Fig. S4C). Gene Ontology (GO) and Kyoto Encyclopedia of Genes and Genomes (KEGG) enrichment pathways analysis suggested that PS treatment influenced the GPCR signaling and cAMP signaling pathway that acts directly on PKA [28] (Fig. S4D and Fig. 6E). Based on gene set enrichment analysis (GSEA), GO and KEGG analyses also suggested that PS treatment up-regulated adenylate cyclase (AC)-activating GPCR signaling pathway and oxidative phosphorylation in adipocytes (Fig. 6F and G). In addition, cAMP-PKA signaling-related genes and ACs, including *Adcy5*, *Adcy6, Adcy3, Adcy2,* and *Adcy7*, were significantly up-regulated (Fig. 6H and Fig. S4E). Consequently, we examined the expression of genes that encoded ACs in adipocytes and adipose tissue of mice with or without PS treatment. As the expression of *Adcy3* and *Adcy7* was elevated under PS treatment (Fig. 6I and J), we further investigated their expression at the protein level and found that ADCY3 was significantly increased due to PS treatment (Fig. 6K to N and Fig. S4F and G). Thus, we speculate that ADCY3-cAMP-mediated signaling may be responsible for PS-induced thermogenic activation. To test this hypothesis, we measured intracellular levels of cAMP and found that PS treatment significantly increased cAMP concentrations in differentiated adipocytes and adipose tissue of mice (Fig. 6O to R). To determine the role of ADCY3 in the PS-related activation of the cAMP-PKA-PGC1α signaling pathway, we performed gain-of-function and loss-of-function experiments (Fig. 6S to U and Fig. S4H). Compared with the control (GFP) cells, 3T3-L1 cells that were overexpressing ADCY3 (OE, *Adcy3*) showed higher levels of cAMP due to PS (Fig. 6S). In contrast, knockdown (KD, sh-*Adcy3*) of ADCY3 robustly blocked the PS-related elevation of intracellular cAMP concentration and the expression of mitochondrial function-related proteins PGC1α (Fig. 6U). Furthermore, cAMP inhibitor SQ22536 (an adenylatecyclase inhibitor) was also used to validate the mechanism. Co-treatment with 10 μM SQ22536 for 3 h partially hindered the activation of PGC1α in the 3T3-L1 cell line that was triggered by PS (Fig. 6V). Collectively, our results indicate that PS-induced up-regulation of ADCY3-cAMP-PKA enhances mitochondrial respiration and PGC1α expression in adipocytes (Fig. 6W).

## Discussion

Our study is the first to demonstrate that “beigeing” fats contain more glycerophospholipids and augment the effectiveness of fat-related KDs in combating obesity. We also identified PS as an important functional glycerophospholipid that contributes to SAT fat KD-related lipid reduction and UCP1 expression in adipose tissue. Mechanistically, exogenous PS enhanced mitochondrial function in adipocytes by activating the ADCY3-cAMP-PKA-PGC1α signaling pathway. Furthermore, the interaction between PS and PGC1α in the NLS region was associated with protein stability and may also play a role in PS-induced thermogenesis. Our study points to health benefits of SAT fat-related KD and further presents a so far unrecognized function of PS in the regulation of adipose tissues thermogenesis. Thus, we propose PS as a future, nonpharmacological tool to treat obesity and its related metabolic diseases (Fig. S5).

As a novel nutritional and dietary approach to counteract obesity, KD consumption has proven effective for the normalization of metabolism and weight loss. Long-term application of KD in DIO mice promotes weight loss and glycemic normalization, and short-term KD application is even more effective than a Mediterranean diet for weight loss ahead of bariatric surgery [29,30]. Fat is the main component of any KD, but only little research has identified the optimal composition of fat (i.e., saturated versus unsaturated, animal fat versus vegetable oil) in a diet for adding health benefits in the treatment of obesity. In this study, we confirmed that a short-term fat-based KD was effective for weight loss in DIO mice. Moreover, a SAT fat-based KD is more likely to promote PGC1α expression and alleviate TC accumulation in the liver and serum than a VAT fat-based KD in DIO mice. In recent years, many studies have been carried out to explore specific characteristics of fat depots with regard to embryonic origin, molecular heterogeneity, metabolic heterogeneity, and secretory roles relating to metabolic diseases [18,31]. Previously, we also found depot-specific differences in fatty acid composition and transcriptional profiles of porcine SAT versus VAT under acute cold exposure [22]. A question that emerged from these studies was whether the heterogeneity in lipid composition of SAT and VAT extends to fat and affects its nutritional properties. Thus, we performed a lipidomic analysis to determine the lipid profiles of fat and found that SAT fat and VAT fat indeed differed in lipid composition.

As we discussed above, adipose tissue is an extraordinarily flexible and heterogeneous organ that is distinct in thermogenic activity [3,32]. Adipose tissue sites with high thermogenic capacity contain more glycerophospholipids than those with low thermogenic capacity [20]. Our previous studies on mouse BAT injury and regeneration models found that glycerophospholipid metabolism plays an important role in the restoration of BAT integrity and homeostasis [33]. A recent study found that inhibition of glycerophospholipid synthesis and transport impaired thermogenesis in adipose tissue [34]. Brown adipocyte-specific deletion of phosphatidylinositol transfer protein results in defective thermogenesis-related metabolism in mitochondria under cold exposure [35]. Glycerophospholipid remodeling has been observed in brown adipocytes and adipocyte mitochondria stimulated by cold or administration of CL-316,243 [36,37]. Hence, we focused on the composition of glycerophospholipids in fats and noticed that SAT fat contained more glycerophospholipids than VAT, especially PG, PI, PS, and PC. The differential glycerophospholipid subclasses in SAT fat and VAT fat might relate to requirements for the induction of the thermogenic program. Cold exposure affects the composition of circulating lipids, inducing distinct increases in PI and PS in mouse plasma and PC and PS in human plasma [25]. This finding is consistent with our previous work demonstrating that PS and PI levels were remarkably increased in the iWAT of UCP1 knock-in pigs [38]. In particular, RNA-seq and lipidomic results have revealed that PS levels are highly correlated with *Ucp1* expression in iWAT [24]. As an important component of the plasma membrane and organelle membrane, most research on PS has focused on its ability to maintain neuronal function, to regulate myoblast fusion and the activity of myomerger protein [39,40]. However, studies relating to the role of dietary PS for lipid physiology still fail to fully elucidate the process. Our findings indicate that PS administration alleviates HFD-induced fat accumulation in the liver. Both short-term and long-term PS intervention could promote weight loss in obese mice. In addition, PS administration enhanced UCP1 expression and mitochondrial function in adipose tissue. Our data suggest that PS is a candidate lipid promoting thermogenesis in adipose tissue.

At the mechanistic level, RNA-seq revealed a subset of genes that were enriched in the cAMP signaling pathway, providing further insights into this signaling pathway. As key cellular signal controlling energy and nutrient homeostasis, cAMP strongly activates PKA [28]. Previous works have demonstrated that lysophosphatidylserine triggers intracellular activation of cAMP-PKA signaling in macrophages [41], and our results confirm that PS, as part of a subclass of glycerophospholipids, can activate cAMP-PKA signaling in adipocytes along with elevating PGC1α and UCP1 expression. AC catalyzes the production of cAMP, and as a key enzyme in the evolutionarily highly conserved cAMP pathway, AC largely regulates organismal physiology during health and disease [42]. ADCY3 has an important role in the regulation of glucose and lipid metabolic homeostasis. In mice, *Adcy3* deficiency leads to impaired insulin sensitivity and dyslipidemia [43], and studies in humans have found that loss-of-function variants in ADCY3 increase the risk for obesity and type 2 diabetes [44]. Seth et al. [45] reported that as potential ligands for ADCYs, 1-stearoyl-2-docosahexaenoyl-phosphatidic acid (SDPA) had increased agonist G protein alpha (Gsα)-mediated activation activity of human ADCY3 7-fold under in vitro conditions. Similarly, we found that PS promoted ADCY3 expression in adipocytes in parallel with an increase in intracellular cAMP concentration. Moreover, we observed that *Adcy3* was knocked down in 3T3-L1 cells and partly blocked PS induced expression of PGC1α, which promotes thermogenic gene expression in adipocytes. Lipids bind to specific protein motifs to modulate protein conformation, and their related functions are involved in several biological processes of cells [46]. A previous study showed that Mfn2 binds to PS and favors the translocation of PS to mitochondria, thereby ameliorating nonalcoholic steatohepatitis in mice [47]. In this study, we found that PS–PGC1α had a stronger binding capacity by using a protein–lipid overlay assay. Focusing on specific binding sites, a previous study identified that cardiolipin binding to 2 lysines (K175 and K269) in the α helices of UCP1 contributed to the structural stability of UCP1 [26]. EPA (5 double bonds) interacts with 3 to 6 hydrophobic residues of GPR120 and the mutations F211^5.42A^ and W207^5.38A^ specifically decreased EPA-induced GPR120 activity [48]. We found that PS interacted with the region (S604 and R608) associated with the stability of the PGC1α protein [27], which suggests that lipid–protein binding may also play a role for PS-related changes in lipid metabolism. The precise role of the interplay between PS and PGC1α, particularly the binding affinity of PS to specific sites on the PGC1α protein, in maintaining the stability of PGC1α, requires further investigation.

In summary, our study compared the dietary effects of SAT- KD and VAT- KD on obesity and characterized the lipid profiles of fats extracted from these 2 adipose tissue sites differing in “beigeing” characteristics. We also investigated the role of the different lipid PS, which closely relate to adipose tissue-related thermogenesis, as potential, dietary lipid for obesity management. We observed that exogenous PS activated UCP1 expression and mitochondrial respiration in adipose tissue. Moreover, we demonstrated that the ADCY3-cAMP-PKA-PGC1α signaling pathway plays a role for PS-induced thermogenesis and that PS–PGC1α binding may facilitate this process by maintaining the stability of PGC1α protein. Our findings provide new insights into the metabolic consequences of lipid compositional heterogeneity and the regulatory role of exogenous PS supplementation for mitochondrial function, suggesting that PS could serve as promising nutritional therapy to counteract obesity and its related metabolic diseases.

## Materials and Methods

### Ethics statement

All experiments with living mice were performed in accordance with the ethical policies and procedures approved by the Animal Care Welfare Committee of Zhejiang University (no. 22703).

### Animals

Male and female C57BL6/J mice were housed in an SPF laboratory under a 12-h light/dark cycle (8 AM to 8 PM) in a humidity- and temperature-controlled environment with free and unlimited access to diet and water.

To activate the DIO model, mice (5 to 6 weeks old) were fed a 60% HFD (Trophic Animal Feed High-Tech Co., Ltd., China) for at least 10 weeks before they exhibited enhanced adiposity. Mice fed a low-fat diet (LFD) (Trophic Animal Feed High-Tech Co., Ltd., China) served as controls. For the KD study, 12 DIO mice were allowed to acclimate to the research facility for 1 week and then were randomly assigned to 2 groups. The HFD was replaced by SAT fat KD or VAT fat KD (Xietong Bioengineering Co., Ltd.), and mice were fed ad libitum for 2 weeks. The diet was changed daily to eliminate possible effects of fat autoxidation. The diet composition is provided in Table S1 [49]. For the diet administration part of the study, 8-week-old mice or DIO mice were kept on a chow diet (Xietong Bioengineering Co., Ltd.) or HFD, respectively. After 1 week of acclimation to the research facility, mice were randomly divided into the PS or control (Con) group (6 mice per group) and then were administered PS by gavage (130 mg kg^−1^ BW per day) or 0.9% NaCl for 7, 14, or 28 consecutive days. The gavage dose of PS was based on the recommended daily intake for adults (500 mg) [50] and the exponent for body surface area (0.67) was used to convert the dose between mice and humans [51]. For the PS intervention study, 5-week-old mice were randomly divided into 2 groups (6 mice per group) and fed an HFD-PS (1% PS instead of 1% fat, referring to the previous studies [52,53]) or HFD for 15 consecutive weeks. The HFD was changed every 3 days. For cold-induced thermogenesis, mice were individually housed in cages at 4 °C in a chamber for 4 h. The dark/light cycle was automatically maintained in the chamber (RDN-type artificial climate chamber, Ningbo Southeast Instrument Co., Ltd). Rectal temperature was measured with a digital display microprobe thermometer at 0, 1, 2, 3, and 4 h. For blood biochemistry measurements, tail blood ketone and glucose concentrations were measured 3 h after feeding (Free Style Optium Neo, Abbott). For glucose tolerance tests (GTTs), mice were injected i.p. with 250 mg ml^−1^ d-glucose (2.5 g kg^−1^ body weight) after overnight fasting (16 h). Blood glucose was measured at 0, 15, 30, 45, 60, 90, and 120 min via the tail vein by a glucometer (Free Style Optium Neo, Abbott). Body composition was analyzed by a Low-field Nuclear Magnetic Small Animal Body Composition Analyser (NIUMAG, Jiangsu, China).

At the end of the experiment, blood samples, adipose tissue, and liver tissue were collected for subsequent analyses.

### Lipid sample preparation and lipidomic assay

Lipid extraction and mass spectrometry-based lipid detection were performed by Applied Protein Technology as previously published [24]. Briefly, a separate sample from each group was taken and mixed together to create a pooled QC sample. QC samples were inserted to test system stability and data reliability in the whole experimental process. LC-MS/MS analysis was performed on a mass spectrometer (Thermo Fisher Scientific, Q Exactive plus) coupled to an ultrahigh-performance liquid chromatography (SHIMADZU, Nexera LC-30A). Full-scan spectra were collected in mass-to-charge ratio (*m*/*z*) ranges of 200 to 1,800 and 250 to 1,800 for positive and negative ion modes, respectively. The *m*/*z* of lipid molecules to lipid fragments was obtained by the following method: after each full scan, 10 fragment patterns (MS2 scan, HCD) were collected. Lipid identification (secondary identification), peak extraction, peak alignment, and quantification were assessed with LipidSearch software version 4.1 (Thermo Scientific). From the extracted ion features, only those variables having more than 50% of the nonzero measurement values in at least one group were considered. A complete list of the lipidomic data is provided in Table S2.

### RNA isolation and quantitative real-time PCR

RNA extraction and quantitative real-time PCR (qPCR) of cells and tissues were performed as previously published [24]. Briefly, total RNA was extracted from adipocytes or adipose tissues using Trizol Reagent (Yeasen Biotechnology [Shanghai] Co., Ltd.), and purity and concentration of total RNA were measured. Two micrograms of total RNA was reverse-transcribed using Hifair III 1st Strand cDNA Synthesis SuperMix (Yeasen Biotechnology [Shanghai] Co., Ltd.). Real-time PCR was carried out with Bio-Rad CFX Connect using SYBR Green Master Mix (Roche) and gene-specific primers. The 2^−ΔΔCT^ method was used to analyze the relative changes in gene expression normalized against 18S ribosomal RNA as an internal control. Primers used for qPCR are shown in Table S3.

### Protein extraction and Western blot analysis

Total protein was isolated from cell or tissue samples using RIPA buffer (Fude Biological Technology Co., Ltd.) supplemented with protease and phosphatase inhibitor cocktails (Thermo Fisher Scientific). Protein separation and Western blot analysis were conducted as described earlier [54]. Immunodetection was performed using enhanced chemiluminescence Western blotting substrate (Biosharp) and detected by ChemiScope 6000 (Shanghai Qinxiang Scientific Instrument Co., Ltd). Specific protein bands were quantified using ImageJ software (v 1.53k). The antibodies used are provided in Table S4.

### Cell culture and adipogenic differentiation

Primary SVF cells were isolated using collagenase digestion followed by density separation as previously published [54]. Briefly, the adipose tissue was minced and digested for 0.5 (BAT) or 1 hour (iWAT) in digestion buffer (1.5 mg/ml collagenase in 1 × phosphate-buffered saline [PBS]) at 37 °C within a shaking water bath. The digestion was terminated with Dulbecco’s modified Eagle’s medium (DMEM) (Sigma, USA) containing 10% fetal bovine serum (FBS) (Gibco, CA, USA) and filtered through a cell strainer (70 μm, Biologix, USA) to remove undigested trunks of tissues and connective tissues. The filtered SVFs were centrifuged at 450*g* for 5 min to separate the SVF cells and then seeded and cultured in growth medium (DMEM containing 20% FBS and 1% penicillin/streptomycin) at 37 °C with 5% CO_2_ for 2 days, and the medium was changed every 2 days. For SVF cell adipogenic differentiation, cells were induced with induction medium (IM) containing DMEM, 10% FBS, 2 μg/ml insulin, 0.25 mM dexamethasone (DEXA), and 0.5 mM 3-isobutylmethylxanthine (IBMX) for 4 days and then differentiated in differentiation medium (DM) containing DMEM, 10% FBS and 2 μg/ml insulin for 2 days until adipocytes matured. Cells were induced to differentiate at 90% confluence. For PS storage solution preparation, PS was dissolved in DMSO at a concentration of 10 mM. For use, PS storage solution was diluted in culture medium or DMEM at a ratio of 1:1,000. For SQ22536 storage solution preparation, SQ22536 was dissolved in DMSO at a concentration of 10 mM.

### Immunofluorescence staining

White adipose preadipocytes or brown adipose preadipocytes, cultivated on glass coverslips, were fixed in 4% paraformaldehyde fix solution for 10 min. Cells were incubated with blocking buffer containing 5% goat serum, 2% BSA, and 0.2% Triton X-100 in PBS for 1 h. Then, the samples were incubated with primary antibodies overnight at 4 °C. After washing with PBS 3 times, the samples were incubated with secondary antibodies for 45 min at room temperature. Nuclei were exposed by incubating samples for 10 min with DAPI. Fluorescence images were captured as single-channel grayscale images using a Leica DM 6000B fluorescence microscope with a 20× objective (NA 0.70). Antibodies are shown in Table S4.

### Molecular identification of PS and PGC1α

Model building, energy minimization, and model evaluation: The complete sequence of the mouse PGC1α protein structure was obtained from the National Center of Biotechnology Information database. Subsequently, the Alpha Fold program [55] was used to conduct ab initio modeling of the PGC1α protein for subsequent molecular recognition studies. The completed and optimized protein model was evaluated by the PROCHECK program. Establishment and optimization of the PGC1α–PS docking model: The molecular structure of the substrate was obtained through PubChem. The MOPAC program [56] was used to optimize the molecular structure and calculate the atomic charge of PM3. The initial structure of PGC1α and PS was processed with AutoDock Tools 1.5.6 [57] to preserve the original charge and generate a pdbqt file for docking. The software package AutoDock 4.2.6 was used for molecular docking, the center coordinates of the docking box were set as (5.321, 24.930, 23.991), the number of cells in each direction of XYZ was set as 60×60×60, the number of docking times was set as 100, and the other parameters were set as default values. The energy optimization method Amber14 force field was used to release these forces and make them more stable structures.

### RNA-seq analysis

RNA-seq of PS-treated white adipose preadipocytes was performed by Sangon Biotech (Shanghai, China). Sequencing libraries were generated from 1 μg of total RNA using the NEBNext UltraTM RNA Library Prep Kit for Illumina (NEB, USA), following the manufacturer’s recommendations. The libraries were then quantified and pooled. Paired-end sequencing of the library was performed on HiSeq XTen sequencers (Illumina, San Diego, CA). FastQC (version 0.11.2) was used to evaluate the quality of the sequenced data. Gene expression data of the transcripts were computed by StringTie (version 1.3.3b). TPM values were used to eliminate the influence of gene lengths and sequencing discrepancies to compare gene expression between samples directly. Differential expression analysis of 2 groups was performed using the DESeq2 R package (1.16.1). Genes were considered as differentially expressed if they met the following criteria: *P* value < 0.01, *q* value < 0.05, and |fold change| > 1.5.

### Pathway enrichment analysis

GSEA was conducted using the clusterProfiler package (version 4.8.3) [58]. The fold change of gene expression was calculated, and the gene list was generated according to the change of |log2FC|. GO analysis was performed through the gseGO function in the clusterProfiler package. The adjusted *P* value < 0.05 was set as the cutoff criteria. KEGG pathway enrichment analyses were also conducted by the gseKEGG function in clusterProfiler package. The adjusted *P* value < 0.05 was set as the cutoff criteria. Functional enrichment analyses, including GO and KEGG analyses, were performed using the OmicStudio tools at https://www.omicstudio.cn/tool. The top 20 GO terms and top 30 KEGG pathways are shown.

### Data analysis

For quantitative analyses, a minimum of 3 biological replicates were analyzed. For all bar graphs, the data are presented as the means ± SEM. Comparisons were performed using 2-sided Student’s *t* test, or one-way analysis of variance (ANOVA) with Tukey’s test. Calculations were performed using GraphPad Prism (v.9.0.0). *P* < 0.05 was considered to indicate significance.

## Data Availability

All data are available from the corresponding author upon request.
