# Supplementary material for: Dietary “Beigeing” Fat Contains More Phosphatidylserine and Enhances Mitochondrial Function while Counteracting Obesity
Source: Research (Wash D C). 2024 Sep 26;7:0492. doi: 10.34133/research.0492 (PMC11425158; doi:10.34133/research.0492)
Supplement: Supplementary 1 — Figs. S1 to S5 Tables S1 to S4 Supplementary methods [file research.0492.f1.zip › Supplemental information.docx]

**Support Information**

**Full title:** Dietary “Beigeing” Fat Contains More Phosphatidylserine and Enhances Mitochondrial Function While Counteracting Obesity

**Short title:** Phosphatidylserine Protects Against Diet Induced Obesity

Yanbing Zhou ^1, 2, 3^, Defeng Ling ^1, 2, 3^, Liyi Wang ^1, 2, 3^, Ziye Xu ^1, 2, 3^, Wenjing You^1, 2, 3^, Wentao Chen^1, 2, 3^, Qiuyun Nong ^1, 2, 3^, Teresa G. Valencak^1^, Tizhong Shan ^1, 2, 3*^

* Address correspondence to: Tizhong Shan; tzshan@zju.edu.cn

**Supplementary methods**

**Serum biochemistry**

Mouse blood samples were obtained from the retro-orbital veins. The samples were placed in sterile Eppendorf (EP) tubes (1.5 ml) for 1 hour and then centrifuged (2000 r × 5 min), and the upper serum was collected to perform blood biochemistry assays. The levels of TG, TC, HDL, LDL and glucose were determined by an automatic biochemical analyzer.

**H&E and immunohistochemistry staining**

Adipose tissue samples were fixed in 4% formalin at room temperature for 24 h, embedded in paraffin and cut at into 4-µm thick slices. Sections were dewaxed, rehydrated, and stained. For H&E staining, the nuclei were stained with hematoxylin for 15 min, rinsed in running tap water and stained with eosin for 1 min. For immunohistochemistry staining, a primary antibody against UCP1 (Abcam) was diluted in 3% BSA and applied to the slides overnight at 4 °C. Then, the primary antibody was washed away using PBS and 3% BSA. Diluted anti-rabbit secondary antibodies conjugated to horseradish peroxidase (HRP) were applied for 60 minutes at RT. After gradient ethanol dehydration, the slides were stabilized with mounting medium. Whole-slide digital images were collected with a Pannoramic MIDI scanner (3DHISTECH Ltd).

**TG and TC measurement**

TG and TC levels in cell or tissue samples were measured using a High Fatty Sample Triglyceride Content Assay Kit (Applygen Technologies Inc.,China) and High Fatty Sample Total Cholesterol Content Assay Kit (Applygen Technologies Inc.) according to the instruction manual. Results are expressed in mmol TG or TC per g protein.

**Surface heat measurement**

Thermal images of mice were acquired by a FLIR E8 infrared camera (used for thermometry) approximately 35 cm away from mice and the temperature data for each group were statistically analyzed. T-tests were used to compare changes in surface temperature between the two groups.

**Oil Red O staining**

Cultured cells were washed with PBS and fixed with 4% formaldehyde for 15 min at room temperature. Then, cells were stained using Oil Red O working solutions containing Oil Red O stock solution (3 g l^−1^ in 60% isopropanol) for 20 min. After staining, the cells were washed with 60% isopropanol in PBS and pictured. Oil Red O dye was extracted from stained adipocytes with 100% isopropanol, and the Oil Red O signal was quantified by measuring the optical density at 490 nm (OD 490)**.**

**Mito-tracker and Nile red staining**

Cultured cells were incubated with 20 nM MitoTracker® Red CMXRos (Invitrogen) for 15 minutes. Intracellular lipids were visualized by staining with 0.5 nM Nile red (Solarbio) for 10 min. Then, the cells were washed with PBS 3 times, fresh DMEM medium was added and pictures were taken.

**Cellular respiration measurements**

Cellular respiration was measured using an Agilent Seahorse XFe96 Extracellular Flux Analyzer (Agilent). In brief, mitochondrial stress tests were performed according to a manufacturer-recommended protocol (Agilent). For differentiated primary adipose cells, after 24 hours of incubation with PS or DMSO (10 μM，DMEM，FBS free ), a standard Seahorse basal medium (Agilent) supplemented with glucose (10 mM), pyruvate (1 mM), and glutamine (2 mM) as substrates was utilized, and oligomycin (15 μM), FCCP (5 μM), and antimycin A/rotenone (5 μM) were added. Oxygen consumption rates (OCRs) and extracellular acidification rates (ECARs) were measured by an Agilent Seahorse XFe96 Extracellular Flux Analyzer following the instrument.

Lipid protein overlay assay

Lipids were immobilized on a PVDF membrane, incubated with the protein of interest and then detected by western blotting as previously published 41. The lipids used in the test were PS (Macklin) and phosphatidylcholine (PC) (Sangon Biotech). **Lipid protein overlay assay**

Lipids were immobilized on a PVDF membrane, incubated with the protein of interest and then detected by western blotting as previously published [1]. The lipids used in the test were PS (Macklin) and phosphatidylcholine (PC) (Sangon Biotech).

**cAMP quantification**

The total concentration of cellular cAMP and was analyzed using the cAMP Direct Enzyme Immunoassay Kit (Enzo Life Sciences). For adipose tissue samples: the snap frozen tissues were weighed and added 10X the volume of 0.1M HCl. The sample was homogenized using a Polytron-type homogenizer at -10℃ for 1 min at 30 Hz. Then, the sample was centrifuged at 12000 rpm for 10 minutes, the supernatant was collected into a new EP tube and this step was repeated twice to remove the lipids. The sample was kept on ice for immediate use or stored at -80°C.For cell samples, the supernatants of adipocytes were removed and treated with 0.1 M HCl for 10 min at room temperature. Cells were scraped off the surface and collected into 1.5 ml EP tubes and then centrifuged at 12000 rpm to pelletize the cellular debris. The supernatant was assayed immediately according to the manufacturer’s instructions.

**Cell transfection, plasmids, and RNA knockdown**

*Adcy3* overexpression plasmid were purchased from Vigene Biosciences (Shandong, China). For *Adcy3* knockdown, the following shRNA sequences were used:

sh*Adcy3*-1：5'GATCCGATCTTTCCCAGGTCATTTCTCCGAGAAATGACCTGGGAAAGATGGCtttttt3'

sh*Adcy3*-2：5'GATCCGCCTCTACCTGTGTGCTATCATCTCGAGATGATAGCACACAGGTAGAGGTTTTT3'

The specificity and efficiency of the *Adcy3* knockdown plasmids were evaluated by western blotting. Among them, the sh*Adcy3*-2 had the best knockdown efficiency and was used for subsequent experiments.

Reference

1. Susila, H., S. Juric, K. Gawarecka, K.S. Chung, S. Jin, S.J. Kim, et al., *In vitro Assays to Evaluate Specificity and Affinity in Protein-phospholipid Interactions.* Bio Protoc, 2022. 12(10), e4421 DOI: 10.21769/BioProtoc.4421.

**Figures and Legends**

**
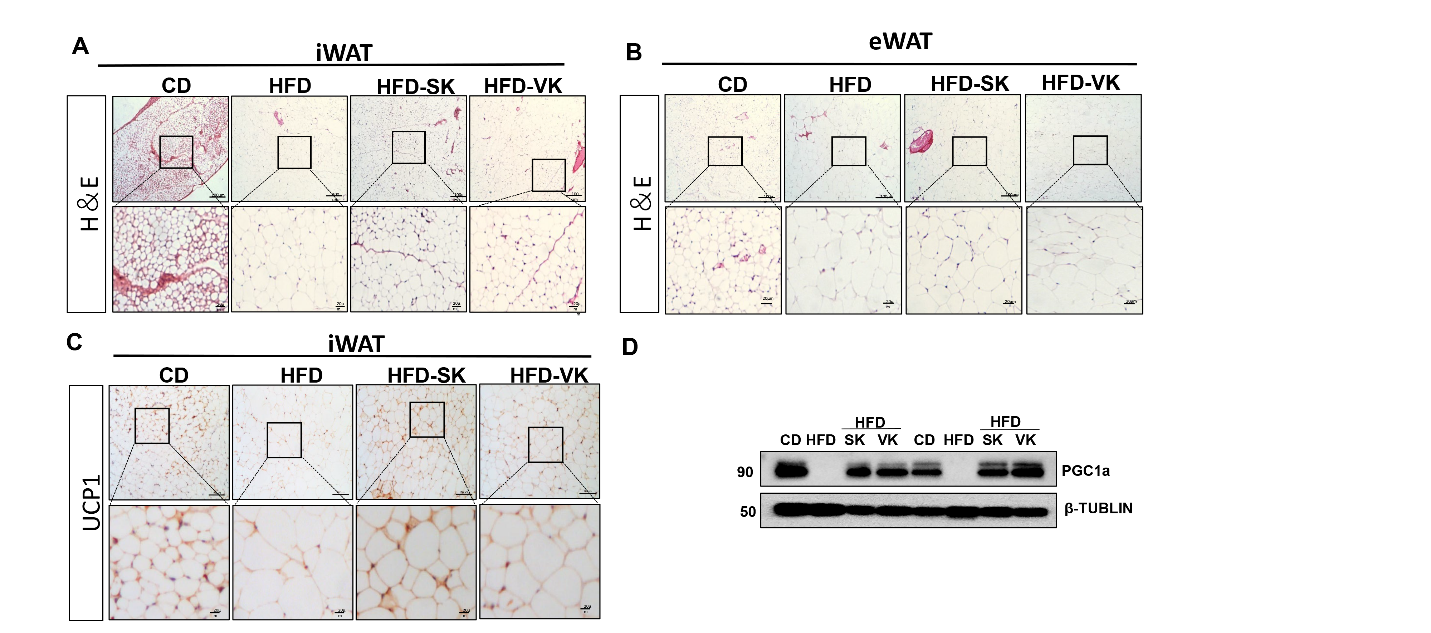
**

**Supplementary figure 1. KD effectively reduced lipid accumulation in adipose tissue of DIO mice (related to Figure 1)**. (A) Representative H＆E staining of iWAT and (B) eWAT from four groups. Scale bars, 100 mm. (C) Representative UCP1 immunostaining of iWAT from four groups. Scale bars, 100 mm. (D) Protein levels of PGC1α in iWAT.

**
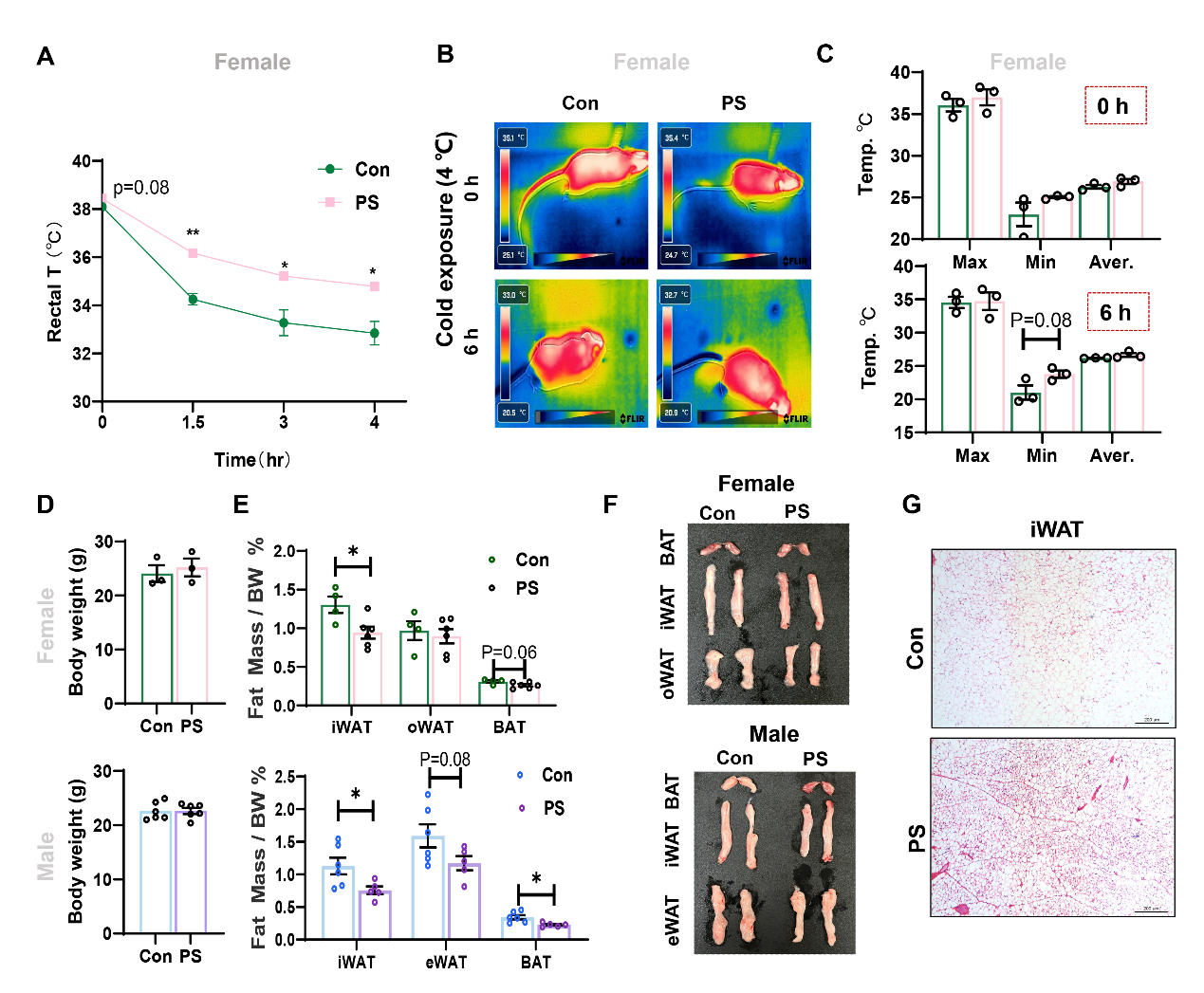
**

**Supplementary figure 2. Administration of PS for 7 consecutive days increased the ability of mice to maintain body temperature during acute cold exposure (related to Figure 2)**. (A) Rectal temperature of female mice in the Con and PS group after cold exposure. (B) Thermal imaging of mice before (0 h) and after cold exposure (6 h). C Maximum, minimum and average surface heat of PS mice and Con mice before (0 h) and after (6 h) exposure to cold. (D) Body weights of mice after 1 week PS or saline administration. (E) Weights of adipose tissue. The error bars represent SEM. **P* < 0.05, ***P* < 0.01, two-tailed Student’s t test. (F) Representative macroscopic images of adipose tissue of mice after 7 days. (G) Representative H & E staining of iWAT of female mice. Scale bars, 200 μm.


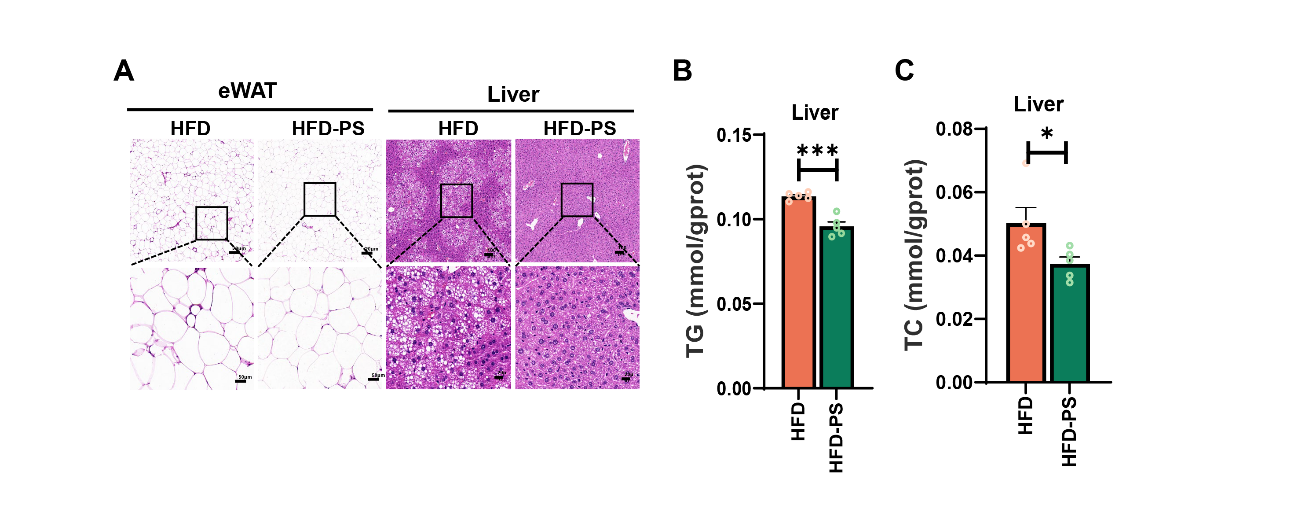


**Supplementary figure 3.** **PS attenuated HFD-induced lipid deposition in eWAT and liver (related to Figure 3)**. (A) Representative H & E staining of eWAT and liver sections. The scale bar is marked on the picture. (B) TG and (C) TC contents in mouse livers (n=4). The data are presented as means ± SEM. *P < 0.05, **P < 0.01, two-tailed Student’s t test.


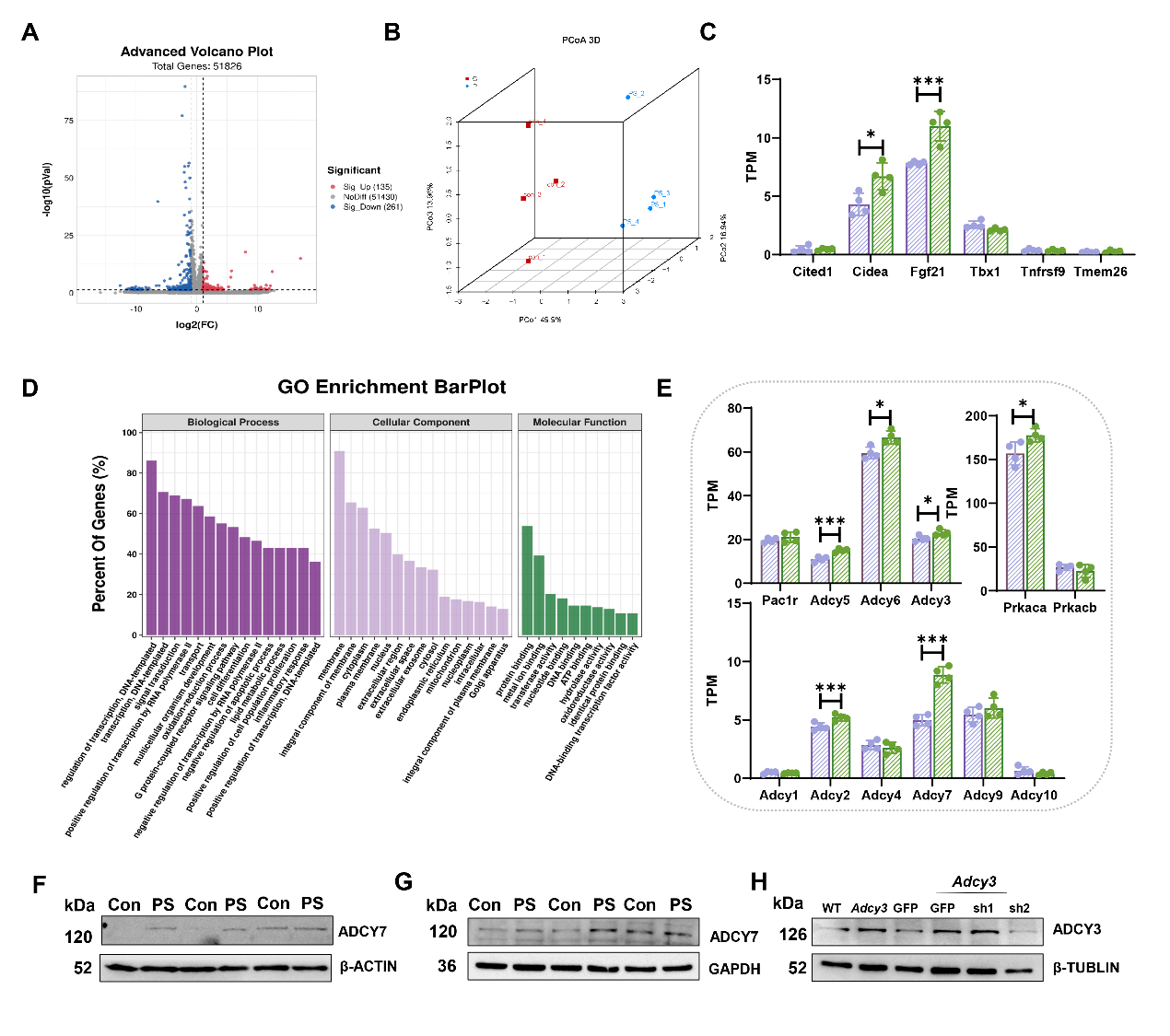


**Supplementary figure 4. PS promoted the expression of *Adcys* in white adipocytes (related to Figure 6).** (A) Volcano plot of differentially expressed gene (DEG) expression patterns in white adipocytes treated with PS. Red denotes upregulated genes in the PS group; blue denotes downregulated genes in the PS group; gray denotes genes with no significant changes. (B) principal coordinate analysis score plot comparing Con with PS samples. Red and blue symbols represent Con and PS samples, respectively. (C) TPM expression values of selected beige adipocyte marker genes from the RNA-seq dataset. (D) Gene Ontology (GO) enrichment analysis. (E) TPM expression values of selected genes relating to cAMP and PKA signals from the RNA-seq dataset. (F) Expression of ADCY7 protein in white and (G) brown adipocytes after 24 hours treatment with PS. (H) ADCY3 expression in Adcy3-OE (represented as Adcy3) or Adcy3-KD (represented as sh1 and sh2) HEK-293T cells 24 hours after plasmid transfection. The data are presented as means ± SEM (n = 4). *P < 0.05, two-tailed Student’s t test.

**
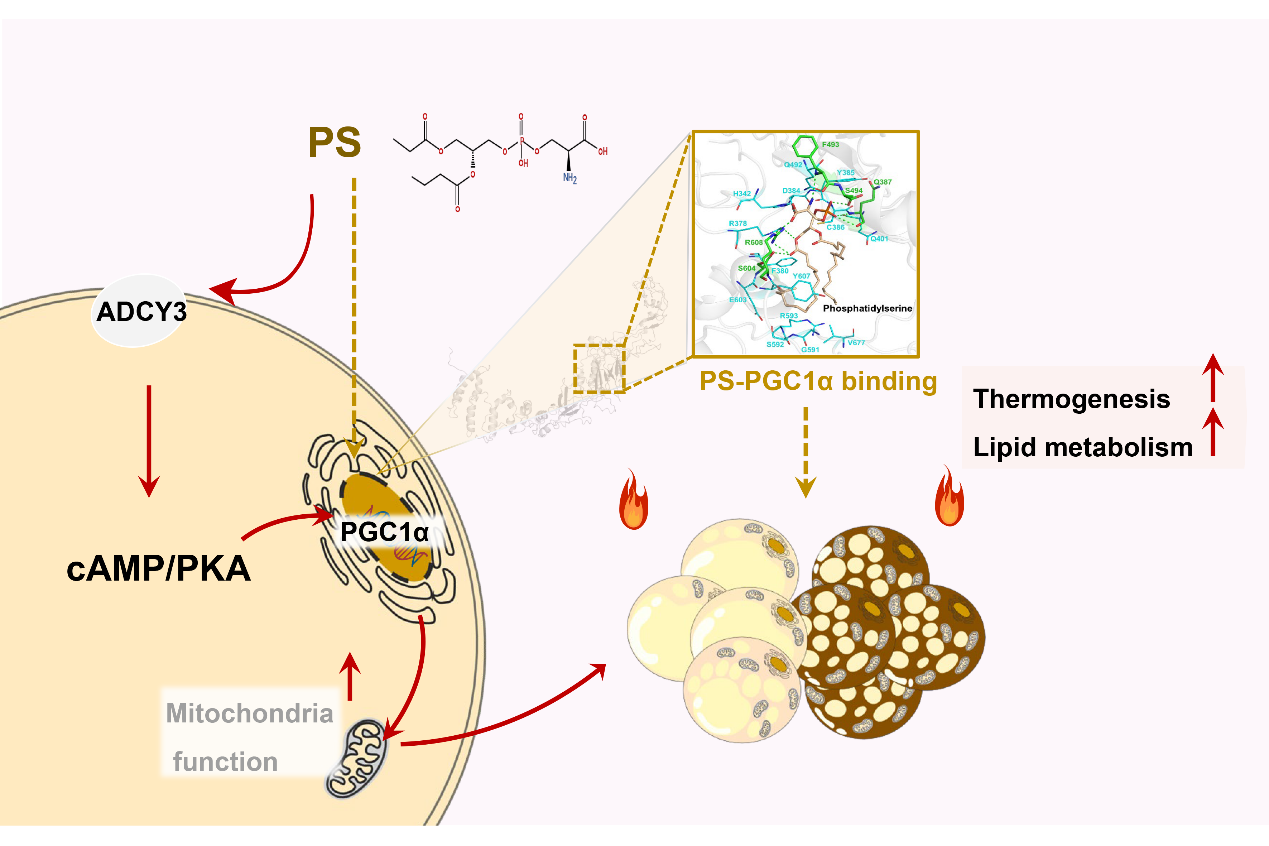
**

**Supplementary figure 5. Diagram of the working model.** PS supplementation leads to an upregulation of *Adcy3* expression and an increase in intracellular cAMP levels. As a result, the PKA-PGC1α pathway is activated, which further upregulates the expression of PGC1α, thereby promoting and induces thermogenesis in adipocytes. Which further promotes UCP1 expression and mitochondria function, thereby inducing thermogenesis in adipocytes.

**Supplementary Tables**

**Supplementary Table 1 Fat-based ketogenic diet**

| Ingredient (g/kg diet） |  |
| --- | --- |
| Protein, of which | 183.7 |
| Casein | 181 |
| L-cystein | - |
| D-methionine | 2.7 |
| Carbohydrates, of which | - |
| Corn starch | - |
| Maltodextrin | - |
| Sucrose | - |
| Fat, of which | 631 |
| Soybean oil | 70 |
| Lard | 561 |
| Choline bitartrate | ++ |
| Cellulose(73.5mg/day) | 85 |
| Tert-butylhydroquinone | 0.126 |
| Mineral mix | 60 |
| Vitamin mix | 13 |
| Other minerals | 27.5 |

**Supplementary Table 2 Mass spectrometry lipid detection data** （Presented in a separate excel）

**Supplementary Table 3 Primers used for qPCR.**

| Gene | Primer Name | Primer Sequence (5′-3′) |
| --- | --- | --- |
| 18s | Forward | AGTCCCTGCCCTTTGTACACA |
|  | Reversed | CGATCCGAGGGCCTCACTA |
| Adcy2 | Forward | CACAGCGCTGGTCACTGGGG |
|  | Reversed | CCGGGCCTCCTTGGACACCT |
| Adcy3 | Forward | CCGTTCCTGCAGACTCTCTC |
|  | Reversed | CCACGCAACTCTGGGTCTAT |
| Adcy5 | Forward | CTTGGGGAGAAGCCGATTCC |
|  | Reversed | ACCGCTTAGTGGAGGGTCT |
| Adcy6 | Forward | TGAGTCTTCTAGCCAGCTCTG |
|  | Reversed | CAGCACCAAGTAGGTGAACCC |
| Adcy7 | Forward | GTGCTGGTGTATGTCGAGTG |
|  | Reversed | GCCTAGTACCATGAGGCAAGC |

**Supplementary Table 4 Key reagent or resource**

| **Reagent or resource** | | **Source** | | **Identifier** |  |  |
| --- | --- | --- | --- | --- | --- | --- |
| **Antibodies** | |  | |  |  |  |
| Anti-ATP5A1 | | Proteintech | | Cat#14676-I-AP |  |  |
| Anti-Beta Actin | | HuaBio | | Cat# B4-B2 |  |  |
| Anti-Cocktail | | Thermo Fisher Scientific | | Cat#45-8099 |  |  |
| Anti-FABP4 | | HuaBio | | Cat#E71703-98 |  |  |
| Anti-GAPDH | | HuaBio | | Cat#EM1101 |  |  |
| Anti-PPARα | | Affinity | | Cat#AF5301 |  |  |
| Anti-Perilipin-1 | | Cell Signaling Technology | | Cat#D1D8 |  |  |
| Anti-phospho-PKA (Thr198) | | Affinity | | Cat#AF7246 |  |  |
| Anti-Protein kinase A (PKA) | | Affinity | | Cat#AF7746 |  |  |
| Anti-Beta Tublin | | HuaBio | | Cat# EM1701-59 |  |  |
| Anti-UCP1 | | Abcam | | Cat#ab10983 |  |  |
| Anti-PGC1α | | ABclonal | | Cat#A20995 |  |  |
| Anti-ATGL | | Huabio | | Cat# RT1058 |  |  |
| Anti-ADCY3 | | HuaBio | | Cat#ER1903-37 |  |  |
| Anti-ADCY7 | | HuaBio | | Cat#ER1903-39 |  |  |
| Goat Anti-Mouse IgG (H+L)-HRP Conjugate | | Invitrogen | | Cat#A28177 |  |  |
| Goat Anti-Rabbit IgG (H+L)-HRP Conjugate | | Invitrogen | | Cat#A27036 |  |  |
| Goat Anti-Rabbit IgG H&L (Alexa Fluor® 647) | | Abcam | | Cat#ab150079 |  |  |
| **Cell lines** | | | | |  |  |
| Mice white preadipocytes | | This study | | N/A |  |  |
| Mice brown preadipocytes | | This study | | N/A |  |  |
| 3T3-L1 | | ATCC | | N/A |  |  |
| Hek-293 | | ATCC | | N/A |  |  |
| **Cell culture reagents** | | | | |  |  |
| Bovine serum albumin (BSA) | Sangon Biotech | | Cat# A600332 | |  |  |
| Dulbecco’s Modified Eagle’s Medium-high Glucose (DMEM) | | Gibco^TM^ | | Cat#11995040 |  |  |
| Phosphate buffer solution (PBS) | | Servicebio | | Cat#G4202 |  |  |
| Collagenase I | | Gibco^TM^ | | Cat#17100017 |  |  |
| Fetal bovine serum (FBS) | | Gibco^TM^ | | Cat# 10099141 |  |  |
| Penicillin/streptomycin | | Sangon Biotech | | Cat#E607011-0100 |  |  |
| Trypsin EDTA | | Sigma-Aldrich | | Cat#T4049 |  |  |
| Insulin solution human | | Sigma-Aldrich | | Cat#I9278 |  |  |
| Dexamethasone (DEX) | | Sangon Biotech | | Cat#A601187-0005 |  |  |
| 3-isobutyl-methylxanthine (IBMX) | | Sangon Biotech | | Cat#A606630-0100 |  |  |
| **Chemicals** | |  | |  |  |  |
| TRIzoL | | Invitrogen | | Cat#15-596-018 |  |  |
| PS | | Macklin | | Cat#S832149 |  |  |
| PC | | Sangon Biotech | | Cat#A510030 |  |  |
| Oil Red O | | Sangon Biotech | | Cat#A600395-0050 |  |  |
| Nile Red | | Solarbio | | Cat#N8440 |  |  |
| Mito-Tracker Red CMXRos | | Beyotime | | Cat#C1049B |  |  |
| SQ22536 | | Selleck | | Cat#S8283 |  |  |
| **Critical commercial assays** | |  | |  |  |  |
| First Strand cDNA Synthesis Kit | | Thermo Fisher Scientific | | Cat#K1631 |  |  |
| XF Cell Mitochondrial Stress Test Kit | | Agilent | | Cat#103015-100 |  |  |
| XF DMEM Base medium | | Agilent | | Cat#103575-100 |  |  |
| Seahorse XFe96 FluxPak mini | | Agilent | | Cat#102601-100 |  |  |
| High Fatty Sample Triglyceride Applygen  Content Assay Kit | | | | Cat# E1025-105 |  |  |
| High Fatty Sample Total Cholesterol Content Assay Kit | | Applygen | | Cat# E1026-105 |  |  |
| cAMP direct Enzyme Immunoassay Kit | | Enzo Life Sciences | | Cat#ADI-900-067A |  |  |
| **Animals food** | |  | |  |  |  |
| 60% high fat diet | | Trophic Diet | | Cat# TP 23300 |  |  |
| Low fat diet | | Trophic Diet | | Cat# TP 23302 |  |  |
| Lard-based KD | | Xietong Bio-engineering CO., LTD | | N/A |  |  |
| Chow diet | | Xietong Bio-engineering CO., LTD | | Cat#SWS9102 |  |  |
| **Western blot reagents** | |  | |  |  |  |
| RIPA buffer | | Fude | | Cat#FD009 |  |  |
| PVDF membrane | | Millipore | | Cat#ISEQ00010 |  |  |
| Protease inhibitor | | Thermo Fisher Scientific | | Cat#A32963 |  |  |
| Pierce BCA assay kit | | Thermo Fisher Scientific | | Cat#23225 |  |  |
| **Deposited data** | |  | |  |  |  |
| *Mice white preadipocyte RNA-Seq data* This study N/A | | | | |  | N/A |
| mass spectrometry lipid detection data in Table S4 | | This study | | N/A |  |  |
| **Software and algorithms** | |  | |  |  |  |
| ImageJ v1.52a | | NIH | | RRID:SCR_003070 |  |  |
| Seahorse Wave Software | | Agilent | | N/A |  |  |
| Microsoft Excel | | Microsoft | | RRID:SCR_016137 |  |  |
| GraphPad Prism 8.3.0 | | Graphpad | | RRID:SCR_002798 |  |  |
| Adobe Photoshop (CS6) | | Adobe | | RRID:SCR_014199 |  |  |
